# Two Cases of Cardiovascular Adverse Events Following Subcutaneous Vosoritide Injection in Early Infancy

**DOI:** 10.7759/cureus.59695

**Published:** 2024-05-05

**Authors:** Atsushi Nishioka, Natsuho Adachi, Hiroyuki Tanaka, Yoichiro Oda

**Affiliations:** 1 Department of Pediatrics, Chigasaki Municipal Hospital, Chigasaki, JPN; 2 Department of Pediatrics, The University of Tokyo, Tokyo, JPN

**Keywords:** c-type natriuretic peptide, pediatric, infant, compensated shock, hypotension, adverse event, cardiovascular, vosoritide, achondroplasia

## Abstract

Achondroplasia, characterized by short stature and skeletal abnormalities, is caused by a gain-of-function variant in the fibroblast growth factor receptor 3 gene. Vosoritide, a C-type natriuretic peptide analog, is an emerging treatment for achondroplasia that functions by promoting endochondral ossification. Vosoritide was approved for the treatment of achondroplasia in Europe and the United States in 2021, and in Japan, the following year. However, vosoritide is associated with a risk of hypotension and vomiting after subcutaneous injection due to its vasodilating effect. Herein, we present two cases of cardiovascular adverse events in infants following vosoritide injection. Case 1 involved a one-month-old female infant with achondroplasia who received the first subcutaneous injection of vosoritide 30 minutes after her last formula intake. Following injection, she developed transient symptomatic hypotension accompanied by vomiting. Although established guidelines recommend that injections be administered after approximately 30 minutes (Europe/Japan) or within one hour (USA) following the last feeding, an extended interval of 1.5 to two hours was required to prevent hypotension-associated vomiting. Case 2 involved a three-month-old female infant with achondroplasia. The first subcutaneous vosoritide injection was administered four hours after the last formula intake, and she subsequently developed prolonged compensated shock with marked tachycardia requiring intervention, including repetitive bolus saline injection. These cases indicate the need to monitor patients for cardiovascular adverse events following subcutaneous injection of vosoritide in early infancy.

## Introduction

Achondroplasia, a genetic disease caused by a gain-of-function variant of the fibroblast growth factor receptor 3 (*FGFR3*) gene, is characterized by markedly short stature, shortened limbs, peculiar facial features, foramen magnum occipitalis stenosis, sleep apnea, middle ear dysfunction, spinal canal stenosis, and spinal kyphosis. An associated gain-of-function variant of *FGFR3* suppresses endochondral ossification and inhibits long tubular bone growth [1]. Vosoritide, an analog of the human C-type natriuretic peptide (CNP), inhibits the signaling pathways downstream of *FGFR3*, thus promoting endochondral ossification and the growth of long bones in achondroplasia [1-3].

In 2019, the results of the first international clinical trial of vosoritide in patients with achondroplasia aged five to 14 years were published [2]. In children with achondroplasia, once-daily subcutaneous administration of vosoritide resulted in a sustained increase in the annualized growth velocity for up to 42 months. In 2021, vosoritide was approved in Europe for the treatment of patients aged two years or older with achondroplasia accompanied by unclosed epiphyseal lines [4], and in the United States, for the treatment of the same patient group aged five years or older [5]. In 2022, in Japan, vosoritide was approved for patients in whom the epiphyseal plate was not closed, but without a minimum age limit, despite a lack of published reports on young patients. Subsequently, in 2023, approval of vosoritide was extended to patients four months of age and older in whom the epiphyseal plate was not closed in Europe, and to patients of all ages in whom the epiphyseal plate was not closed in the United States.

Herein, we present two cases of cardiovascular adverse events following vosoritide injection in one- and three-month-old infants.

## Case presentation

Case 1

**Figure 1:**
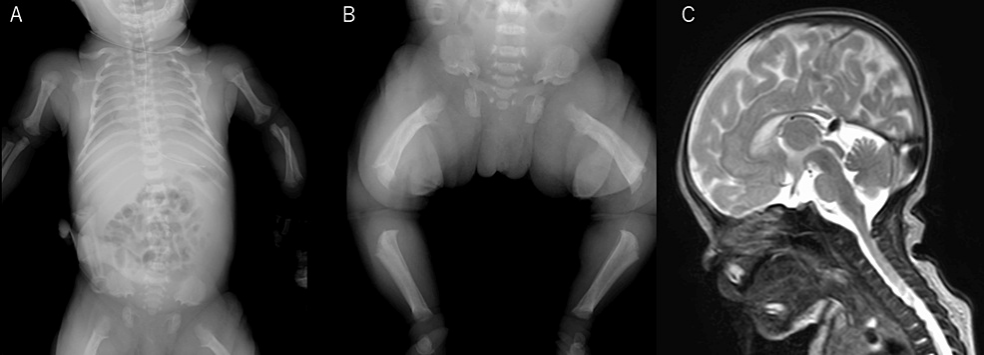
Clinical features of case 1. (A) X-ray image of the body at birth showing short, robust tubular bones, squared-off iliac wings, flat acetabula, marked narrowing of the sacrosciatic notch, and a characteristic proximal femoral radiolucency. (B) X-ray image of the legs at birth showing the same finding as in image A. (C) MRI at eight days of age showing mild foramen occipitalis magnum stenosis.

Case 1 involved a female infant born to healthy, unrelated Japanese parents. Fetal echocardiography had indicated shortening of the femur length and abnormal bone shape, potentially indicating achondroplasia. She had been born via induced vaginal delivery at 38 weeks and four days of gestation. Her birth weight, height, and head circumference were 2,981 g (0.2 SD), 45.0 cm (-1.80 SD), and 35.5 cm (1.4 SD), respectively. Physical examination revealed macrocephaly, midfacial hypoplasia, a small chest, short fingers in trident configuration, and disproportionate short stature with rhizomelic limb shortening. Radiography revealed short, robust tubular bones, squared-off iliac wings, flat acetabula, marked narrowing of the sacrosciatic notch, and a characteristic proximal femoral radiolucency (Figures 1A, 1B). At eight days of age, head magnetic resonance imaging (MRI) revealed mild foramen occipitalis magnum stenosis (Figure 1C). No apnea or vomiting was observed. Next-generation sequencing, performed by Kazusa DNA Research Institute (Chiba, Japan) [6], identified a heterozygous p.Gly380Arg variant of *FGFR3*, confirming the diagnosis of achondroplasia.

Vosoritide injection was initiated at one month and 23 days of age, when the patient had a body weight of 4,305 g and a body length of 51.1 cm. The first vosoritide dose of 0.12 mg was injected subcutaneously 30 minutes after formula feeding. However, 20 minutes after the injection, the patient developed a pallid complexion and lethargy and began vomiting; her blood pressure, measured using an automatic sphygmomanometer, decreased to 56/29 mmHg, and hypotension was confirmed through multiple measurements. No injection-site reactions, exanthema, or other symptoms indicative of drug allergy were observed. Thirty-five minutes after injection, the patient recovered from hypotension without any intervention (Table 1). The second subcutaneous injection of vosoritide (0.12 mg) was administered 30 minutes after formula feeding and a similar course of transient symptomatic hypotension and vomiting was observed. The third injection, comprising a reduced dose of 0.08 mg, was administered one hour after feeding, and hypotension was not observed. Self-injection of vosoritide at home was subsequently initiated at a dose of 0.08 mg. Two weeks later, the dose was increased to 0.12 mg, without recurrence of hypotensive symptoms. Two months later, owing to weight gain, the dose was again increased from 0.12 mg to 0.16 mg to maintain the 30-µg/kg dosage. After this increase in dosage, she developed transient hypotensive symptoms with vomiting 20-30 minutes after the injection several times a month, and the parents were eventually obliged to extend the interval between formula intake and injections to 1.5 to two hours to prevent vomiting. Vomiting only occurred after vosoritide injections. At eight months of age, she no longer presented with any hypotensive symptoms, including vomiting; thus, the parents reverted to feeding formula 30 minutes or one hour before injections, without any symptom recurrence.

**Table 1:** Clinical course of the two infant cases BP, blood pressure (mmHg); P, pulse; SpO2, percutaneous oxygen saturation (%); BS, blood glucose concentration (mg/dL); IV, intravenous injection; × 1, once; × 2, twice.

Case	Vosoritide injection	Time (minutes)	Event
				Case 1	1st injection	-30	Last formula intake
		0	Vosoritide subcutaneous injection 0.12 mg (30 μg/kg). BP = 99/46
		20	Pallid complexion, lethargy, vomiting. BP = 56/26, P = 90, SpO2 = 100
		35	Ruddy complexion, active. BP = 87/47, P = 128, SpO2 = 98%
	2nd injection	-30	Last formula intake
		0	Vosoritide subcutaneous injection 0.12 mg (30 μg/kg). BP = 83/45
		10	Pallid complexion. BP = 63/43, P = 144
		25	Pallid complexion, lethargy, vomiting. BP = 83/43, P = 156
		35	Ruddy complexion, active
Case 2	1st injection	-240	Last formula intake
		0	Vosoritide subcutaneous injection 0.16 mg (30 μg/kg). BP = 94/66
		28	Pallid complexion, yawning, cold sweats. BP = 106/78, P = 180–190, SpO2 = 99–100, BS = 105
		45	160 mL of formula intake, ruddy complexion, vigorous. P = 180–190, SpO2 = 99, BS = 138
		60	Pallid complexion, lethargy. BP = 135/47, P = 176, SpO2 = 99, BS = 156
		70	Pallid complexion, lethargy. Saline IV × 2 (total 30 mL). P = 160→130
		75	Saline IV × 1 (5 mL), 20% glucose IV × 1 (5 mL). BP = 146/66, P = 160→150
		96	Ruddy complexion, no lethargy
		110	Ruddy complexion, active. P = 150, SpO2 = 100

Case 2

**Figure 2:**
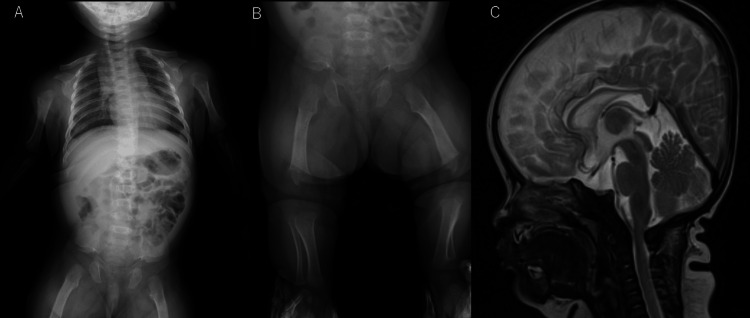
Clinical features of case 2. (A) X-ray image of the body at two months and 12 days of age showing short, robust tubular bones, squared-off iliac wings, flat acetabula, marked narrowing of the sacrosciatic notch, and a characteristic proximal femoral radiolucency. (B) X-ray image of the legs at two months and 12 days of age showing the same finding as in image A. (C) MRI at two months and 12 days of age showing mild foramen occipitalis magnum stenosis.

Case 2 involved a female infant born to unrelated Japanese parents. The mother had already been diagnosed with achondroplasia before pregnancy. Fetal echocardiography indicated shortening of the femur and humerus length. The infant was born at 39 weeks and 0 days of gestation. Her birth weight, height, and head circumference were 2,598 g (-0.82 SD), 45.5 cm (-1.70 SD), and 35.0 cm (1.38 SD), respectively. Physical examination at age two months and 12 days revealed macrocephaly, midfacial hypoplasia, small chest, short fingers in trident configuration, and disproportionate short stature with rhizomelic limb shortening. Radiography revealed short, robust tubular bones, squared-off iliac wings, flat acetabula, marked narrowing of the sacrosciatic notch, and a characteristic proximal femoral radiolucency (Figures 2A, 2B). Head MRI indicated mild foramen magnum occipitalis stenosis (Figure 2C). No apnea or vomiting was observed. Next-generation sequencing, performed by Kazusa DNA Research Institute (Chiba, Japan) [6], identified a heterozygous p.Gly380Arg variant of *FGFR3*, confirming the diagnosis of achondroplasia.

The first subcutaneous vosoritide injection was administered four hours after formula feeding at a dose of 0.16 mg at three months and 19 days of age when the patient had a body weight of 5,160 g and a body length of 54.7 cm. Twenty-eight minutes after the injection, the infant developed a pallid complexion, accompanied by yawning and cold sweats. No injection-site reactions, exanthema, or other symptoms indicative of drug allergy were observed. Although her blood pressure, measured using an automatic sphygmomanometer, was 106/78 mmHg, reactive tachycardia with a pulse rate ranging from 180 to 190 beats/minute was observed. Her blood glucose level was 105 mg/dL. Forty-five minutes after the injection, the patient’s condition improved with formula feeding. However, 60 minutes after the injection, she again developed a pallid complexion, lethargy, and tachycardia (pulse rate of 176 beats/minute) without hypotension. An intervention comprising repetitive bolus saline administration totaling 30 mL improved tachycardia (from 160 to 130 beats/minute). Her complexion was gradually improved. Finally, 110 minutes after the vosoritide injection, she returned to activity with a ruddy complexion (Table 1). The patient’s family refused further vosoritide treatment.

Informed consent was obtained from the parents of the two patients for the publication of this case report.

## Discussion

Herein, we presented two cases of cardiovascular adverse events in infants following vosoritide injection. In case 1, transient symptomatic hypotension with vomiting developed after a subcutaneous injection of vosoritide, with prompt recovery. Initially, vomiting was prevented by extending the interval between formula intake and vosoritide administration from 30 minutes to one hour prior to injection. Later, this interval required an extension of 1.5 to two hours to prevent vomiting after injections. Notably, vomiting was not observed, except after injections, indicating a relationship between treatment and vomiting. In case 2, the injection was administered four hours after the last feeding, resulting in compensated shock with marked tachycardia. Although the patient’s condition temporarily improved with formula feeding, 60 minutes after injection, a pallid complexion, lethargy, and tachycardia without hypotension recurred, requiring intervention. The tachycardia improved with repetitive administration of a saline bolus, suggesting that the cause of the tachycardia was hypoperfusion. Indeed, it should be noted that the interval between the final feeding and the injection was longer than that specified in the instruction manual provided by the Japanese manufacturers (Table 2) [5]. Nevertheless, cardiovascular adverse events were strong and persistent, compared with those in previous reports [3,7]. Owing to the short half-life of subcutaneously injected vosoritide, cardiovascular adverse events should be transient, with full recovery generally achieved within 60 minutes. The biphasic course of case 2 could be interpreted as an effect of increased blood flow to the gastrointestinal tract due to formula ingestion.

**Table 2:** Manufacturer’s instructions for vosoritide administration in the US, EU, and Japan US, the United States; EU, European Union.

Source	Country	Description
The vosoritide website for U.S. patients [8]	US	To reduce the risk of a decrease in blood pressure and associated symptoms (dizziness, feeling tired, or nausea), patients should eat a meal and drink eight to 10 ounces of fluid within one hour before receiving Voxzogo.
Summary of product characteristics [9]	EU	Patients should be well hydrated at the time of injection. It is recommended that patients eat a light snack and drink an adequate amount of fluid (e.g., water, milk, and juice) about 30 minutes before injecting. This is to reduce the signs and symptoms of potential decreases in blood pressure (dizziness, fatigue, and/or nausea).
Vosoritide injection instructions for healthcare providers [5]	Japan	Transient hypotension or symptoms associated with hypotension (dizziness, nausea/vomiting, fatigue, syncope, etc.) may occur. Patients should be instructed to take appropriate fluid intake (e.g., a light meal or a glass of water (water, milk, juice, etc.) approximately 30 minutes before administration). (In Japanese, author’s translation)

Hypotension after vosoritide injection is a concern owing to the vasodilatory effects of CNP. In an early international trial in patients with achondroplasia aged five to 14 years, hypotension and vomiting were observed in 16/35 (46%) and 11/35 (31%) patients, respectively [2]. In a later study, Chan et al. found no clinically meaningful correlations between plasma exposure to vosoritide and changes in pre-dose pulse rate or systolic or diastolic blood pressure in patients with achondroplasia treated with vosoritide [7]. In a recent international vosoritide phase II trial investigating vosoritide use in younger patients with achondroplasia aged three to 59 months, mild, transient, and asymptomatic decreases in systolic or diastolic blood pressure were observed in both the vosoritide and placebo groups [3]. However, one of the 43 (2.3%) patients who received vosoritide injections further experienced a symptomatic hypotensive event that resolved without intervention. This trial included achondroplasia patients at a younger age than those in the first trial by Savarirayan et al. [2]. Indeed, the youngest cohort (cohort 3) included nine patients aged three to six months. However, owing to the two-month observation period after enrollment, the mean (±SD) age at the initiation of vosoritide injection was 5.66 ± 0.44 months; our two patients were even younger. We are not sure whether the very young age of our patients contributed to the risk of cardiovascular adverse events. Owing to the small number of patients in this age group, additional cases are needed to properly investigate adverse events.

The package insert for vosoritide lists hypotension and vomiting as possible side effects. To prevent hypotension following injections, the instructions for patients in the US provided by the manufacturer on the vosoritide website recommend eating and drinking 8-10 ounces of fluids within one hour before receiving the injection [8], whereas the corresponding instructions in the European Union [9] and Japan [5] recommend having a light snack and drinking an adequate amount of water, milk, or juice approximately 30 minutes before receiving the injection (Table 2). However, the rationale behind these different recommendations is unclear. Adequate fluid intake prior to the injection has been indicated as being beneficial to prevent hypotension after injection, as it can help prevent orthostatic hypotension by fluid intake [10]. In contrast, hypotension with a full stomach increases the risk of vomiting, as observed in case 1. The gastric emptying time has been well-studied owing to the risk of aspiration pneumonia associated with vomiting during general anesthesia [11-13]. Recent guidelines from the European Society of Anesthesiology recommend adherence to the 6-4-3-1 rule (six hours: food, four hours: formula/snack, three hours: breast milk, one hour: clear water) as a fasting regimen before the induction of anesthesia [14]. Furthermore, an international multidisciplinary consensus statement on fasting before procedural sedation recommends following the 4-2-0 rule (four hours: formula or food fluid, two hours: breast milk, no limit: clear water) for infants aged <12 months [15].

Overall, our experience with these two cases indicates that clinicians should consider the risk of cardiovascular adverse events following vosoritide injection in early infancy. The interval between the final fluid intake and injection was too short in case 1 and too long in case 2. Therefore, the optimal interval between adequate fluid intake and vomiting should be considered as a trade-off. In early infancy, it may be advantageous to extend the interval between the final formula intake and the vosoritide injection to, for example, 1.5 to two hours. Further investigation is required to confirm this.

## Conclusions

In this study, we reported two cases of female infants, aged one and three months, who developed cardiovascular adverse events following vosoritide injection. Although the recommended interval between the last formula feeding and vosoritide injection has been variably defined as 30 minutes (in Europe and Japan) or within one hour (in the United States) in different instruction manuals, case 1 required an extended interval of 1.5 to two hours between the last formula intake and injection to prevent hypotension-induced vomiting. This experience suggests that the interval should be carefully adjusted based on individual responses to minimize the risk of cardiovascular adverse events following the subcutaneous injection of vosoritide in patients in early infancy.
